# Survival effects of postoperative adjuvant TACE in early-HCC patients with microvascular invasion: A multicenter propensity score matching

**DOI:** 10.7150/jca.87435

**Published:** 2024-01-01

**Authors:** Junlin Qian, Yanling Shen, Lifeng Cui, Zhao Wu, Shuju Tu, Wei Lin, Hongtao Tang, Zemin Hu, Liping Liu, Wei Shen, Yongzhu He, Kun He

**Affiliations:** 1 Department of Hepatobiliary Surgery, Zhongshan People's Hospital (Zhongshan Hospital Affiliated to Sun Yat-sen University), Zhongshan City, Guangdong Province, China 528400.; 2 Division of Hepatobiliary and Pancreas Surgery, Department of General Surgery, The First Affiliated Hospital of Nanchang University (The First Clinical Medical College of Nanchang University), Nanchang City, Jiangxi Province, China 330006.; 3 Division of Hepatobiliary and Pancreas Surgery, Department of General Surgery, Shenzhen People's Hospital (The Second Clinical Medical College, Jinan University; The First Affiliated Hospital, Southern University of Science and Technology), Shenzhen City, Guangdong Province, China 518020.; 4 Department of General Surgery, The Second Affiliated Hospital of Nanchang University (The Second Clinical Medical College of Nanchang University), Nanchang City, Jiangxi Province, China 330006.; 5 Maoming People's Hospital, Maoming City, Guangdong Province, China 525000.

**Keywords:** Microvascular invasion, Hepatocellular Carcinoma, Transarterial chemoembolization, Hepatectomy

## Abstract

**Background:** The presence of microvascular invasion (MVI) significantly worsens the surgical outcome of hepatocellular carcinoma (HCC). The purpose of this research was to investigate the survival benefit of adjuvant transarterial chemoembolization (TACE) in patients with MVI after hepatectomy.

**Methods:** A retrospective analysis was conducted on 1372 HCC patients who underwent curative liver resection in four medical institutions. In order to minimize confounding factors and selection bias between groups, Propensity Score Matching (PSM) (1:1) was performed to ensure balanced clinical characteristics.

**Results:** A total of 1056 patients were enrolled after PSM, including 672 patients with MVI and 384 patients without MVI. Adjuvant TACE improves DFS (Median, 36 months vs 14 months, p < 0.001) and OS (Median, NA vs 32 months, p < 0.001) in patients harboring MVI, but not in those (all p > 0.05) lacking MVI. In different different CNLC stages, adjuvant TACE improved DFS (CNLC stage I, Median, 37 vs 15 months; CNLC stage II, Median, 25 vs 11 months, p < 0.001) and OS (CNLC stage I, Median, NA vs 32 months, p < 0.001; CNLC stage II, Median, NA vs 26 months, p = 0.002) in patients who carried MVI, but not in those (CNLC stage I-II, all p > 0.05) who lacked MVI.

**Conclusions:** Adjuvant TACE may be a potentially effective treatment option for improving survival outcomes in early-HCC patients harboring MVI, but not in those lacking MVI.

## Introduction

Microvascular invasion (MVI) is one of the important risk factors that severely affects the survival outcomes of patients with hepatocellular carcinoma (HCC), and its presence is increasingly recognized as reflecting the increased local infiltration and distant metastasis ability of the tumor 1, 2. Partial hepatectomy and liver transplantation represent potential curative treatments for selected patients with HCC 1-3. Although liver transplantation is far superior to hepatic resection, it is often limited by organ shortage, technical difficulties, and stringent medical conditions 1-5. It can be seen that currently hepatic resection is still the first-line treatment for HCC. Although the effectiveness of hepatic resection has been demonstrated, postoperative recurrence and survival remain the main concerns for HCC patients, especially for those with MVI 3-5.

In recent years, postoperative adjuvant therapy for HCC patients has become a hot topic of concern 6-8. Some high-risk HCC patients may benefit from postoperative adjuvant transarterial chemoembolization (TACE) in clinical practice 8-11. It is particularly worth noting whether patients with MVI can obtain further survival benefits from adjuvant TACE. We evaluated the survival efficacy of adjuvant TACE in MVI patients using clinical data from multiple medical centers, aiming to provide reasonable treatment decisions for clinical work.

## Methods

### Patients

We conducted a retrospective analysis of clinical data from HCC patients at four medical centers between January 2018 and September 2021. Inclusion criteria: (1) All patients underwent liver resection and had confirmed negative surgical margins by pathology; (2) Postoperative pathology confirmed the tumor as HCC; (3) No portal vein tumor invasion, lymph node metastasis, or extrahepatic metastasis were detected. (4) All patients had tumor staging within Chinese Liver Cancer (CNLC) stages I-II.Exclusion criteria: (1) Patients with missing clinical data or incomplete follow-up data; (2) Patients with pathology confirmed as other malignant liver tumors or with a history of other malignancies; (3) Patients who died within 30 days after surgery. The study was performed in accordance with the Declaration of Helsinki and the Ethical Guidelines for Clinical Studies in all participating medical institutions.

### Assessment of MVI and adjuvant TACE

The "7-point" baseline sampling method was used to collect pathological specimens during surgery (**Fig. S1 A**): 1. Samples were collected 1:1 at the junction of cancer and adjacent tissues at 12, 3, 6 and 9 points of the tumor; 2.At least one sample is collected inside the tumor; 3.One piece of liver tissue was taken at a distance of ≤1cm and >1cm from the tumor border, respectively. MVI is defined as the presence of tumor cells in the portal vein, hepatic vein or blood vessels of liver tissue near the tumor margin visible under the microscope (**Fig. S1 BC**).

The risk of recurrence of HCC is evaluated by doctors based on the preoperative clinical data and postoperative pathological indicators of the patient. Patients with a high risk of recurrence (with one or more of the following features: advanced tumor staging, tumor diameter ≥ 5cm, multiple tumors, alpha-fetoprotein (AFP) ≥ 400, microvascular invasion (MVI), satellite nodules and Edmondson-Steiner grade III-IV) are recommended to receive PA-TACE about 4 weeks after hepatectomy. However, patients decided whether or not to follow this recommendation based on their medical compliance, economic status, or other social factors. Prior to receiving adjuvant TACE, patients need to undergo routine examinations such as liver function tests, computed tomography (CT), and/or magnetic resonance imaging (MRI) to confirm good liver function and absence of tumor recurrence. During the operation of TACE, we placed the hepatic arterial catheter through the femoral artery into the proper hepatic artery using the Seldinger technique, and injected a mixture of appropriate chemotherapeutic (Fluorouracil, 400-500 mg/m^2^; Epirubicin, 40-70 mg/m^2^; Lobaplatin, about 50 mg/m^2^) and embolic agents(lipiodol and gelatin sponge, 3-5 mL) through the catheter into the residual liver based on a comprehensive assessment of the patient's body surface area, physical fitness, and residual liver volume 8-11.

### Follow-up

All patients were followed up either through outpatient visits or during hospitalization. Within the first six months postoperatively, patients were followed up approximately every two months, and thereafter, follow-up examinations were conducted approximately every six months. Recurrence was defined as new tumor nodules confirmed by enhanced CT or/and enhanced MRI or needle biopsy. Disease-free survival (DFS) and overall survival (OS) were used as study endpoints. DFS was defined as the time from hepatectomy to diagnosis of tumor recurrence, while OS was defined as the time from hepatectomy to death or the last follow-up. All patients were followed up until April 1, 2022.

### Propensity score matching

The main purpose of propensity score matching (PSM) analysis is to eliminate the imbalance between groups and make the two groups more consistent in other factors except for the intervention, so as to more accurately evaluate the impact of the intervention on the outcome variable. In order to minimize bias between groups, PSM analysis was performed for each subgroup, thus eliminating the imbalance between the subgroups of patients who received or did not receive adjuvant TACE. A 1:1 nearest neighbor matching algorithm was applied with a caliper width of 0.01. SPSS 26.0 statistical software (IBM Corp, Armonk, NY, USA) was used for PSM.

### Statistical methods

Continuous data adhering to a normal distribution were assessed using an independent samples t-test and reported as mean ± standard deviation (SD); Non-normally distributed continuous data were analyzed using the Mann-Whitney U test, and results were reported as median (interquartile range, IQR); Categorical data were examined using the chi-square test, presented as numbers (n) and proportions (%). Cox proportional risk models were utilized for univariate and multivariate analyses, to determine the independent prognostic factors for DFS and OS. In the univariate analysis, variables with a P-value < 0.05 were included in the multivariate analysis. Kaplan-Meier survival analysis was employed to evaluate DFS and OS based on the independent prognostic factors identified, and the differences between the survival curves were assessed using the log-rank test. Statistical analysis of the aforementioned data was performed using R software (Version 4.2.1; http://www.r-project.org). All P-values were calculated using a two-tailed test, and statistical significance was defined as P < 0.05 to indicate significance.

## Results

### Clinical characteristics

This study enrolled a total of 1372 HCC patients, including 815 patients without MVI and 557 patients with MVI. In patients without MVI, 384 received adjuvant TACE, and 431 patients did not receive adjuvant TACE (**Table S1**). Among MVI patients, 328 received adjuvant TACE, and 229 patients did not receive adjuvant TACE (**Table S2**). To more accurately assess the impact of adjuvant TACE on survival outcomes, PSM analysis was performed on subgroups of patients who either received or did not receive adjuvant TACE, within the cohorts of those with and without MVI. After PSM, there were no significant differences in clinical characteristics between groups (**Table 1**, **Table 2**, All p > 0.05).

### Risk factors for survival outcomes

**Figure 1:**
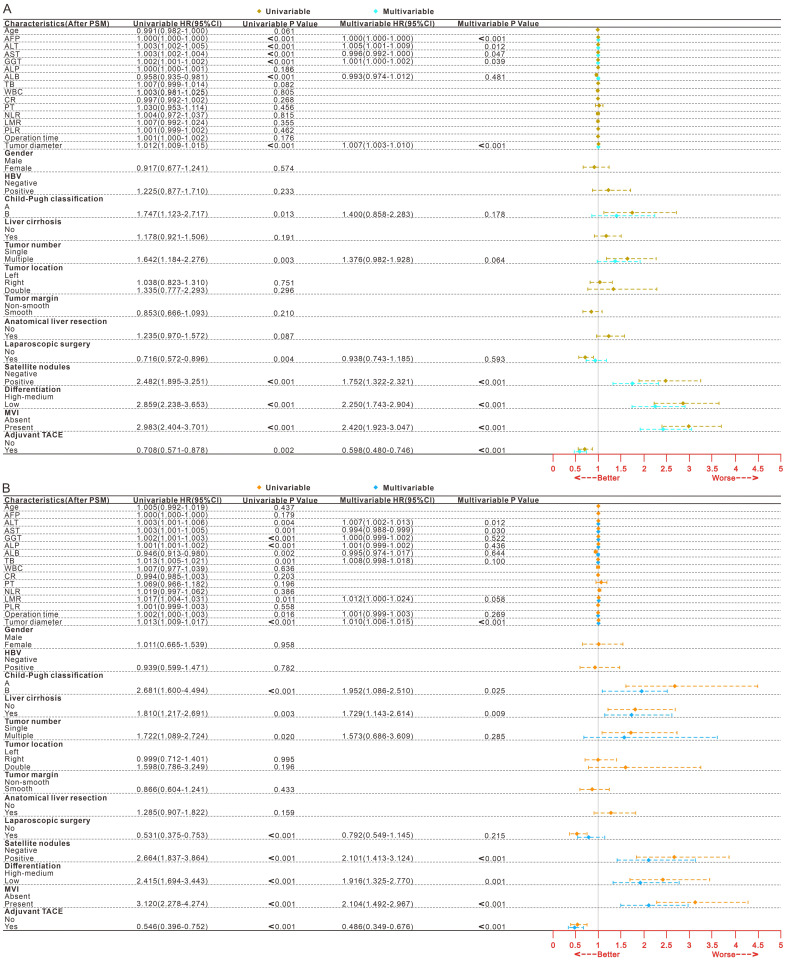
** Forest plot of univariate and multivariate Cox regression analysis of DFS (A) and OS (B) after hepatectomy in HCC patients after PSM.** HCC, Hepatocellular carcinoma; PSM, Propensity score matching; DFS, Disease-free survival; OS, Overall survival; MVI, Microvascular invasion; TACE, Transarterial chemoembolization; HR, Hazard ratio; CI, confidence interval; AFP, Alpha-fetoprotein; ALT, Alanine aminotransferase; AST, Aspartate aminotransferase; GGT, Gamma-glutamyltransferase; ALP, Alkaline phosphatase; Alb, Albumin; TB Total bilirubin; WBC. White blood cell; CR, Creatinine; PT Prothrombin time; NLR, Neutrophil-to-lymphocyte ratio; LMR, Lymphocyte-to-monocyte ratio; PLR, Platelet-to-lymphocyte ration; HBV, Hepatitis B virus

**Figure 2:**
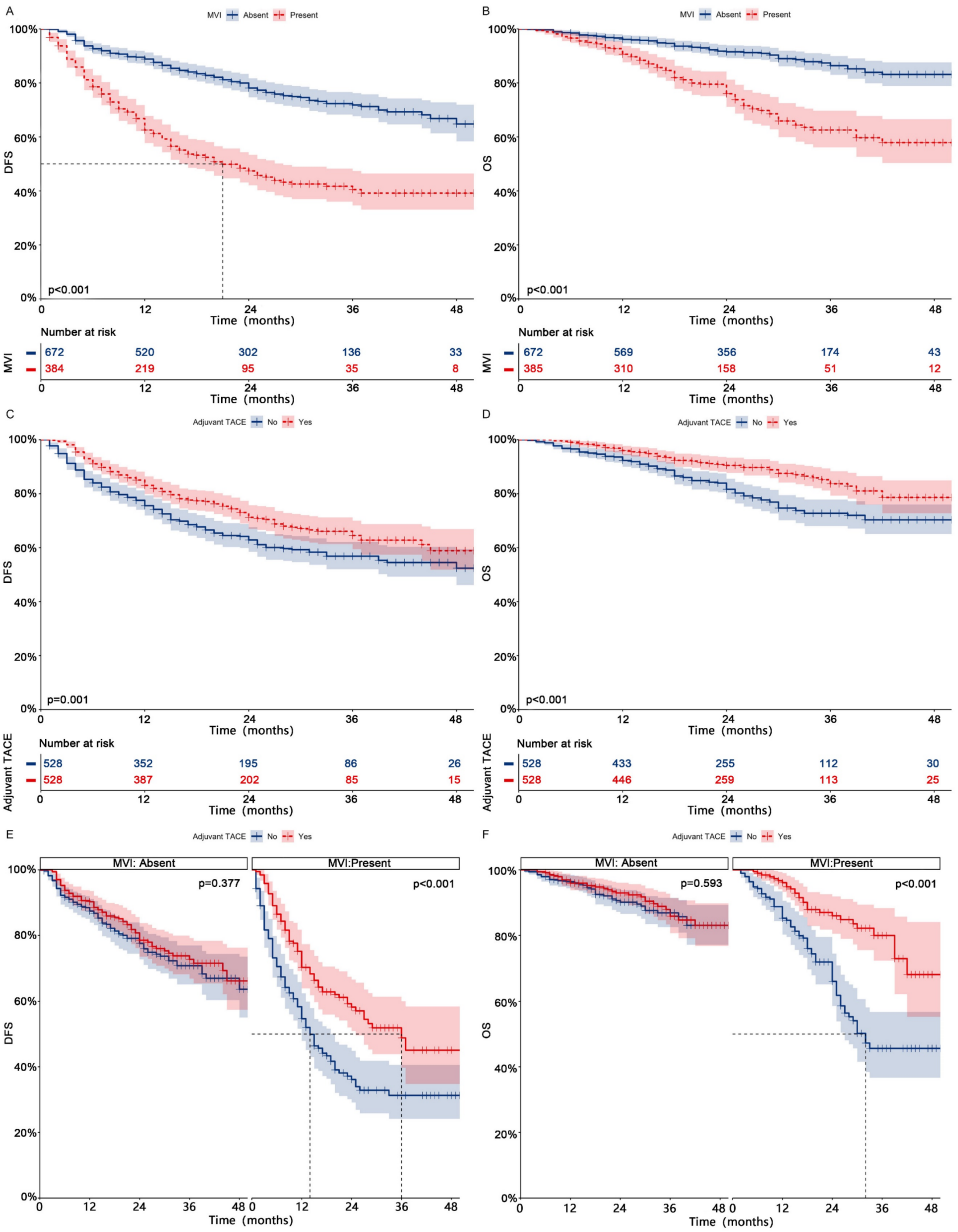
** Kaplan-meier analysis of DFS (A) and OS (B) in HCC patients with or without MVI after PSM; Kaplan-meier analysis of DFS (C) and OS (D) in HCC patients receiving adjuvant TACE or not after PSM; Subgroup Kaplan-meier analysis of DFS (E) and OS (F) in patients with and without MVI receiving adjuvant TACE after PSM.** HCC, Hepatocellular carcinoma; PSM, Propensity score matching; DFS, Disease-free survival; OS, Overall survival; MVI, Microvascular invasion; TACE, Transarterial chemoembolization.

After PSM for all patients, 339 patients experienced tumor recurrence, while 162 patients experienced death (**Table 3,** After PSM; **Table S3,** Before PSM). After univariate and multifactorial Cox regression analysis (**Fig. 1**, After PSM; **Fig.S2**, Before PSM) and Kaplan-Meier analysis (**Fig. 2 ABCD**, After PSM; **Fig. S3 ABCD**, Before PSM), both MVI and non-adjuvant TACE were shown to be risk factors for DFS and OS. Patients receiving adjuvant TACE had significantly higher DFS (83%-71%-65% vs 76%-63%-57%, p = 0.001) and OS (96%-90%-84% vs 92%-82%-73%, p < 0.001) at 1, 2, and 3 years than patients who did not receive adjuvant TACE. Adjuvant TACE improves DFS (Median, 36 months vs 14 months; 1-, 2-, and 3-year, 70%-58%-49% vs 55%-36%-31%, p < 0.001) and OS (Median, NA vs 32 months; 1-, 2-, and 3-year, 96%-86%-80% vs 85%-66%-46%, p < 0.001) in patients harboring MVI, but not in those (DFS, p = 0.377; OS, p = 0.593) lacking MVI (**Fig. 2 EF**, After PSM; **Fig. S3 EF**, Before PSM).

### Subgroup analysis

**Figure 3:**
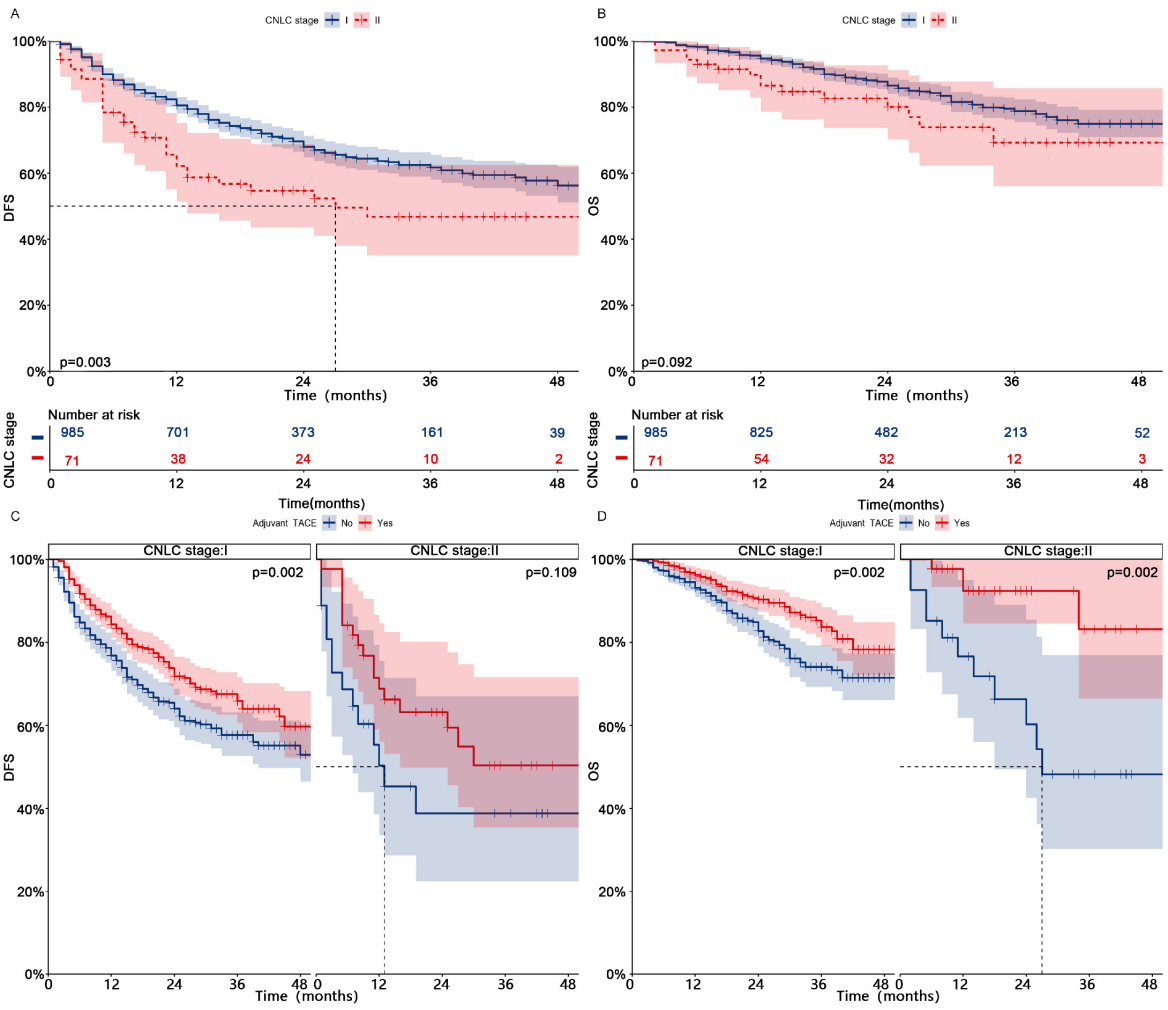
** Kaplan-meier analysis of DFS (A) and OS (B) for patients with different CNLC stages after PSM; Subgroup Kaplan-meier analysis of DFS (C) and OS (D) for patients with different CNLC stages receiving adjuvant TACE after PSM.** PSM, Propensity score matching; DFS, Disease-free survival; OS, Overall survival; TACE, Transarterial chemoembolization; CNLC, China liver cancer

**Figure 4:**
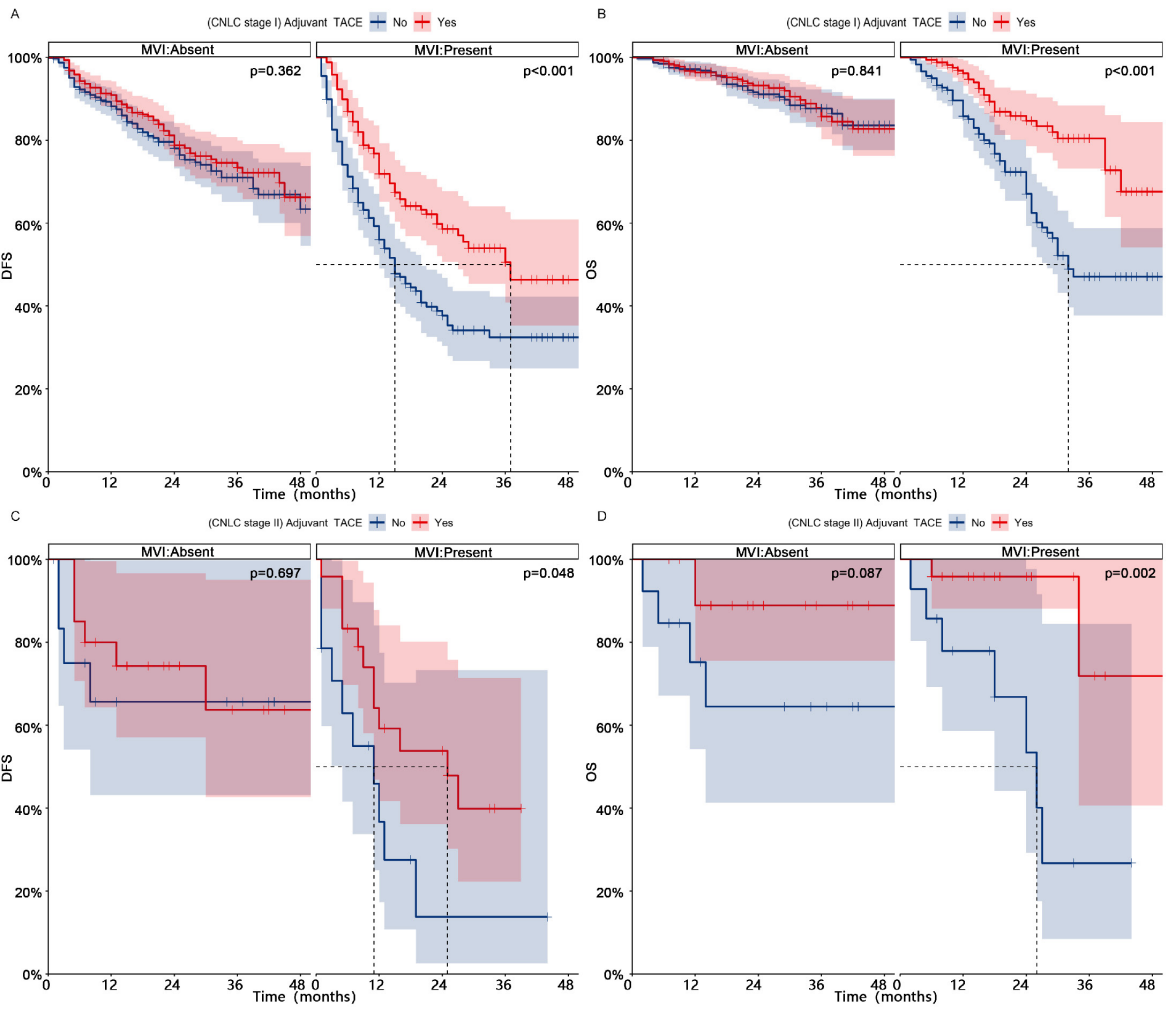
** Subgroup Kaplan-meier analysis of DFS (AC) and OS (BD) for MVI patients in different CNLC stages who received adjuvant TACE after PSM.** PSM, Propensity score matching; DFS, Disease-free survival; OS, Overall survival; MVI, Microvascular invasion; TACE, Transarterial chemoembolization; CNLC, China liver cancer

Patients in CNLC stage I who received adjuvant TACE had significantly higher DFS (1-, 2-, and 3-year, 84%-72%-66% vs 77%-64%-58%, p = 0.002) and OS (1-, 2-, and 3-year, 96%-90%-84% vs 93%-83%-74%, p = 0.002) than those who did not receive adjuvant TACE (**Fig. 3**, After PSM; **Fig. S4**, Before PSM). Patients in CNLC stage II who received adjuvant TACE did not achieve higher DFS (Median, NA vs 13 months; 1-, 2-, and 3-year, 69%-63%-50% vs 50%-39%-39%, p = 0.109), but achieved higher OS (Median, NA vs 27 months; 1-, 2-, and 3-year, 92%-92%-83% vs 77%-60%-48%, p = 0.002). In different different CNLC stages, adjuvant TACE improved DFS (CNLC stage I, Median, 37 vs 15 months, 1-, 2-, and 3-year, 72%-59%-51% vs 56%-38%-32%, p < 0.001; CNLC stage II, Median, 25 vs 11 months, 1-, 2-, and 3-year, 59%-54%-40% vs 37%-14%-14%, p < 0.001) and OS (CNLC stage I, Median, NA vs 32 months, 1-, 2-, and 3-year, 96%-85%-80% vs 86%-67%-47%, p < 0.001; CNLC stage II, Median, NA vs 26 months, 1-, 2-, and 3-year, 96%-96%-72% vs 78%-53%-27%, p = 0.002) in patients who carried MVI, but not in those (CNLC stage I, DFS, p = 0.362, OS, p = 0.841; CNLC stage II, DFS, p = 0.697, OS, p = 0.087) who lacked MVI (**Fig. 4**, After PSM; **Fig. S5**, Before PSM).

## Discussion

MVI typically reveals the high invasiveness and metastatic ability of tumors, and its presence significantly worsens the surgical outcome of HCC 1, 4, 12. Even among patients with tumors < 3 cm in diameter, the incidence of MVI remains over 20% 13, 14. In this study, approximately 40% of HCC patients had MVI detected in their postoperative pathological results, and it was identified as an independent risk factor that significantly affected DFS and OS. Some earlier authors found little benefit from liver transplantation in patients with MVI 4. Reasonable criteria for inclusion of HCC patients in liver transplantation should strike an optimal balance between good surgical outcomes and donor shortage. When both of these procedures are clearly appropriate, liver resection is more appropriate for patients with MVI because the 5-year survival rates are similar for both procedures 4, 5. Thus, MVI is an important pathological examination indicator for evaluating the risk of HCC recurrence and selecting treatment options.

Wang et al. 8 found that the HCC patients with intermediate (tumor size > 5 cm) or high risk of recurrence (single tumor with MVI as well as 2 or 3 tumors) after curative liver resection could benefit from TACE (3-year OS, TACE vs Non-TACE, 85.2% vs 77.4%; P=0.040). Some scholars believe that early recurrence in the remaining liver usually comes from intrahepatic metastasis of the primary tumor, and during the adjuvant TACE period, the combination of embolization agents to block blood supply and local chemotherapy drugs can kill and suppress residual or new tumor cells. Obviously, adjuvant TACE can provide significant survival benefits for MVI patients who are prone to early recurrence 8-11. However, in this study, adjuvant TACE could significantly prolong the survival of patients with MVI, but it was not effective for patients without MVI. A meta-analysis showed that adjuvant TACE not only failed to improve the prognosis of patients without MVI, but also potentially promoted postoperative recurrence in some patients 15. This suggests that adjuvant TACE is not a necessary treatment option for patients without MVI. Therefore, the detection of MVI may help guide the selection of adjuvant TACE after surgery.

There is no unified protocol or standard for the selection and indications of postoperative adjuvant therapy in the international community, and its indications mainly depend on the definition of high-risk recurrence population. Currently, it is widely believed in clinical practice that risk factors affecting early recurrence and survival of patients include tumor diameter, number of tumors, portal vein tumor invasion, and high expression of tumor markers, among others 16, 17. This result is basically consistent with the argument of this research. Interestingly, we found that adjuvant TACE not only prolonged both DFS and OS of the overall patient population, but also resulted in significant survival benefits for patients with different CNLC stages. The reason for this may be related to the possibility that the same patient may have multiple high-risk factors simultaneously. Therefore, other high-risk factors may also help guide the choice of postoperative adjuvant TACE.

The present study has several limitations. First, this research was conducted as a retrospective analysis, which made it impossible to completely avoid patient selection bias. Second, there is still a lack of formal clinical guidelines for postoperative adjuvant TACE, which leads to the possibility that the type and dosage of drugs may vary from one medical center to another. It is hoped that more large, multicenter, prospective trials will emerge in the future to provide more accurate evidence to validate the relevant arguments of this current research.

## Conclusions

In summary, adjuvant TACE significantly improves the survival of early-HCC patients after hepatectomy, especially for MVI patients. However, it has limited efficacy in HCC patients who lack MVI. Overall, adjuvant TACE may be a potential treatment to improve postoperative survival in HCC patients, and the detection of MVI can help guide the choice of postoperative adjuvant TACE.

## Supplementary Material

Supplementary figures and tables.

## Figures and Tables

**Table 1:** Clinical characteristics of patients without MVI who underwent adjuvant TACE or not after PSM

Clinical characteristics		MVI absent			
		Total (n = 672)	Adjuvant TACE		P
			No (n = 336)	Yes (n = 336)	
**Age (years)**		56.00 (48.00, 64.25)	56.00 (47.00, 64.00)	56.000 (49.00, 65.00)	0.518
**AFP (ng/mL)**		22.25 (4.80, 265.73)	20.11 (4.32, 198.15)	25.40 (5.27, 325.98)	0.192
**ALT (U/L)**		30.20 (21.63, 44.00)	29.88 (21.92, 42.64)	31.39 (21.15, 45.00)	0.460
**AST (U/L)**		32.38 (25.45, 44.06)	31.41 (25.00, 43.10)	33.00 (26.08, 45.77)	0.066
**GGT (U/L)**		44.63 (27.00, 79.93)	47.14 (26.11, 79.00)	42.51 (27.00, 80.75)	0.710
**ALP (U/L)**		91.06 (74.00, 117.00)	89.33 (71.00, 112.08)	95.00 (75.00, 120.44)	0.059
**ALB (g/L)**		41.60 (38.70, 44.40)	41.40 (38.70, 43.95)	41.95 (38.65, 44.70)	0.277
**TB (mol/L)**		16.39 (10.73)	16.01 (12.89)	16.78 (8.00)	0.357
**WBC (10^9^/L)**		5.30 (4.25, 6.47)	5.41 (4.26, 6.68)	5.24 (4.21, 6.30)	0.109
**CR (μmol/L)**		73.14 (62.30, 84.02)	73.41 (62.76, 84.42)	73.02 (62.14, 84.00)	0.875
**PT (s)**		11.80 (11.20, 12.50)	11.70 (11.20, 12.40)	11.80 (11.20, 12.60)	0.124
**NLR**		2.10 (1.52, 3.04)	2.06 (1.50, 2.96)	2.17 (1.61, 3.17)	0.165
**LMR**		3.57 (2.70, 4.95)	3.61 (2.73, 5.00)	3.53 (2.69, 4.85)	0.927
**PLR**		103.16 (79.19, 141.24)	97.65 (75.59, 143.19)	107.39 (83.88, 139.58)	0.054
**Operation time (mins)**		210.00 (155.00, 265.00)	200.00 (150.00, 260.00)	211.00 (165.00, 280.00)	0.151
**Tumor diameter (mm)**		35.00 (23.00, 57.00)	35.00 (23.00, 58.00)	34.00 (23.00, 57.00)	0.989
**Gender [n(%)]**	**male**	558 (83.04)	288 (85.71)	270 (80.36)	0.081
	**female**	114 (16.96)	48 (14.29)	66 (19.64)	
**HBV [n(%)]**	**Negative**	94 (13.99)	47 (13.99)	47 (13.99)	1.000
	**Positive**	578 (86.01)	289 (86.01)	289 (86.01)	
**Child-Pugh classification [n(%)]**	**A**	649 (96.58)	324 (96.43)	325 (96.73)	1.000
	**B**	23 (3.42)	12 (3.57)	11 (3.27)	
**Liver cirrhosis [n(%)]**	**No**	194 (28.87)	101 (30.06)	93 (27.68)	0.551
	**Yes**	478 (71.13)	235 (69.94)	243 (72.32)	
**Tumor number [n(%)]**	**single**	626 (93.15)	317 (94.35)	309 (91.96)	0.285
	**multiple**	46 (6.85)	19 (5.65)	27 (8.04)	
**Tumor location [n(%)]**	**left**	214 (31.85)	118 (35.12)	96 (28.57)	0.069
	**right**	439 (65.33)	212 (63.10)	227 (67.56)	
	**double**	19 (2.83)	6 (1.79)	13 (3.87)	
**Tumor margin [n(%)]**	**Non-smooth**	147 (21.88)	70 (20.83)	77 (22.92)	0.576
	**Smooth**	525 (78.12)	266 (79.17)	259 (77.08)	
**Anatomical liver resection [n(%)]**	**No**	217 (32.29)	102 (30.36)	115 (34.23)	0.322
	**Yes**	455 (67.71)	234 (69.64)	221 (65.77)	
**Laparoscopic surgery [n(%)]**	**No**	347 (51.64)	178 (52.98)	169 (50.30)	0.537
	**Yes**	325 (48.36)	158 (47.02)	167 (49.70)	
**Satellite nodules [n(%)]**	**Negative**	621 (92.41)	314 (93.45)	307 (91.37)	0.382
	**Positive**	51 (7.59)	22 (6.55)	29 (8.63)	
**Differentiation [n(%)]**	**High-medium**	592 (88.10)	299 (88.99)	293 (87.20)	0.551
	**Low**	80 (11.90)	37 (11.01)	43 (12.80)	

**PSM**, Propensity score matching; **MVI**, Microvascular invasion; **TACE**, Transarterial chemoembolization; **AFP**, Alpha-fetoprotein; **ALT**, Alanine aminotransferase; **AST**, Aspartate aminotransferase; **GGT**, Gamma-glutamyltransferase; **ALP**, Alkaline phosphatase; **ALB,** Albumin; **TB**, Total bilirubin;** WBC**, White blood cell; **CR**, Creatinine; **PT**, Prothrombin time; **NLR**, Neutrophil-to-lymphocyte ratio; **LMR**, Lymphocyte-to-monocyte ratio;** PLR**, Platelet-to-lymphocyte ratio; **HBV**, Hepatitis B virus

**Table 2:** Clinical characteristics of MVI patients who underwent adjuvant TACE or not after PSM

Clinical characteristics		MVI present			
		Total (n = 384)	Adjuvant TACE		P
			No (n = 192)	Yes (n = 192)	
**Age (years)**		54.50 (46.00, 63.25)	53.00 (45.00, 63.25)	55.000 (46.75, 63.25)	0.365
**AFP (ng/mL)**		103.95 (9.48, 1000.00)	94.15 (7.95, 1000.00)	126.45 (10.65, 1000.00)	0.362
**ALT (U/L)**		30.00 (21.11, 44.12)	27.19 (20.37, 44.60)	30.91 (21.98, 43.87)	0.188
**AST (U/L)**		35.35 (27.00, 52.56)	34.75 (27.00, 53.08)	36.00 (27.00, 52.00)	0.723
**GGT (U/L)**		61.35 (33.00, 118.00)	62.00 (35.00, 118.55)	60.86 (32.00, 111.74)	0.492
**ALP (U/L)**		98.00 (77.93, 126.00)	98.50 (77.20, 128.99)	97.50 (78.00, 123.00)	0.522
**ALB (g/L)**		40.14 (4.27)	40.25 (4.25)	40.04 (4.30)	0.635
**TB (mol/L)**		17.24 (14.92)	17.678 (19.626)	16.806 (7.795)	0.567
**WBC (10^9^/L)**		5.31 (4.34, 6.38)	5.30 (4.38, 6.39)	5.33 (4.30, 6.27)	0.581
**CR (μmol/L)**		72.70 (61.88, 80.46)	73.05 (62.08, 79.75)	71.70 (61.19, 81.78)	0.883
**PT (s)**		11.90 (11.30, 12.60)	11.90 (11.30, 12.50)	11.85 (11.28, 12.73)	0.565
**NLR**		2.31 (1.66, 3.23)	2.40 (1.79, 3.38)	2.18 (1.58, 3.09)	0.140
**LMR**		3.36 (2.50, 4.63)	3.35 (2.50, 4.64)	3.38 (2.49, 4.58)	0.706
**PLR**		112.83 (86.04, 159.95)	111.73 (87.90, 149.18)	115.47 (84.64, 166.37)	0.477
**Operation time (mins)**		230.00 (180.00, 285.00)	230.00 (180.00, 296.25)	225.00 (180.00, 280.00)	0.812
**Tumor diameter (mm)**		50.00 (34.00, 75.25)	48.50 (33.00, 73.00)	51.00 (34.75, 78.50)	0.793
**Gender [n(%)]**	**male**	332 (86.46)	162 (84.38)	170 (88.54)	0.297
	**female**	52 (13.54)	30 (15.62)	22 (11.46)	
**HBV [n(%)]**	**Negative**	52 (13.54)	27 (14.06)	25 (13.02)	0.881
	**Positive**	332 (86.46)	165 (85.94)	167 (86.98)	
**Child-Pugh classification [n(%)]**	**A**	361 (94.01)	177 (92.19)	184 (95.83)	0.197
	**B**	23 (5.99)	15 (7.81)	8 (4.17)	
**Liver cirrhosis [n(%)]**	**No**	88 (22.92)	39 (20.31)	49 (25.52)	0.275
	**Yes**	296 (77.08)	153 (79.69)	143 (74.48)	
**Tumor number [n(%)]**	**single**	335 (87.24)	174 (90.62)	161 (83.85)	0.066
	**multiple**	49 (12.76)	18 (9.38)	31 (16.15)	
**Tumor location [n(%)]**	**left**	122 (31.77)	61 (31.77)	61 (31.77)	1.000
	**right**	242 (63.02)	121 (63.02)	121 (63.02)	
	**double**	20 (5.21)	10 (5.21)	10 (5.21)	
**Tumor margin [n(%)]**	**Non-smooth**	97 (25.26)	52 (27.08)	45 (23.44)	0.481
	**Smooth**	287 (74.74)	140 (72.92)	147 (76.56)	
**Anatomical liver resection [n(%)]**	**No**	95 (24.74)	50 (26.04)	45 (23.44)	0.636
	**Yes**	289 (75.26)	142 (73.96)	147 (76.56)	
**Laparoscopic surgery [n(%)]**	**No**	257 (66.93)	130 (67.71)	127 (66.15)	0.828
	**Yes**	127 (33.07)	62 (32.29)	65 (33.85)	
**Satellite nodules [n(%)]**	**Negative**	307 (79.95)	154 (80.21)	153 (79.69)	1.000
	**Positive**	77 (20.05)	38 (19.79)	39 (20.31)	
**Differentiation [n(%)]**	**High-medium**	307 (79.95)	152 (79.17)	155 (80.73)	0.799
	**Low**	77 (20.05)	40 (20.83)	37 (19.27)	

**PSM**, Propensity score matching; **MVI**, Microvascular invasion; **TACE**, Transarterial chemoembolization; **AFP**, Alpha-fetoprotein; **ALT**, Alanine aminotransferase; **AST**, Aspartate aminotransferase; **GGT**, Gamma-glutamyltransferase; **ALP**, Alkaline phosphatase; **ALB,** Albumin; **TB**, Total bilirubin;** WBC**, White blood cell; **CR**, Creatinine; **PT**, Prothrombin time; **NLR**, Neutrophil-to-lymphocyte ratio; **LMR**, Lymphocyte-to-monocyte ratio;** PLR**, Platelet-to-lymphocyte ratio; **HBV**, Hepatitis B virus

**Table 3:** DFS and OS at 1, 2, and 3 years for different subgroups of population who received adjuvant TACE after PSM

Characteristics [Number (%), Event, Median time (months)]			1 year		2 year		3 year		P
			Adjuvant TACE						
			No	Yes	No	Yes	No	Yes	
**DFS**	**All patients (1056, 339, NA/NA)**		76% (72%-79%)	83% (80%-86%)	63% (58%-68%)	71% (67%-76%)	57% (52%-62%)	65% (59%-70%)	0.001
	**MVI**	**Absent [672 (63.64), 150, NA/NA]**	87% (84%-91%)	90% (87%-94%)	78% (73%-83%)	79% (74%-84%)	71% (65%-77%)	73% (67%-79%)	0.377
		**Present [384 (36.36), 189, 14/36]**	55% (48%-62%)	70% (64%-77%)	36% (29%-45%)	58% (51%-67%)	31% (24%-41%)	49% (40%-60%)	<0.001
	**CNLC stage I (985, 307, NA/NA)**		77% (73%-81%)	84% (81%-88%)	64% (60%-69%)	72% (67%-77%)	58% (53%-63%)	66% (61%-72%)	0.002
	**MVI**	**Absent [639 (64.87), 140, NA/NA]**	88% (85%-92%)	91% (88%-94%)	78% (73%-83%)	79% (74%-84%)	71% (65%-77%)	73% (67%-80%)	0.362
		**Present [346 (35.13), 167, 15/37]**	56% (49%-64%)	72% (65%-79%)	38% (30%-47%)	59% (51%-68%)	32% (25%-42%)	51% (41%-63%)	<0.001
	**CNLC stage II (71, 32, 13/NA)**		50% (33%-76%)	69% (56%-85%)	39% (22%-67%)	63% (50%-80%)	39% (22%-67%)	50% (35%-72%)	0.109
	**MVI**	**Absent[33 (46.48), 10, NA/NA]**	66% (43%-100%)	80% (64%-100%)	66% (43%-100%)	74% (57%-97%)	66% (43%-100%)	64% (43%-95%)	0.697
		**Present[38 (53.32), 22, 11/25]**	37% (17%-77%)	59% (42%-84%)	14% (2.6%-73%)	54% (36%-80%)	14% (2.6%-73%)	40% (22%-71%)	0.048
**OS**	**All patients (1056, 162, NA/NA)**		92% (90%-95%)	96% (94%-98%)	82% (78%-86%)	90% (88%-93%)	73% (68%-78%)	84% (79%-88%)	<0.001
	**MVI**	**Absent [672 (63.64), 66, NA/NA]**	96% (94%-98%)	96% (94%-98%)	90% (87%-94%)	93% (90%-96%)	87% (82%-91%)	86% (81%-91%)	0.593
		**Present [384 (36.36), 96, 32/NA]**	85% (80%-91%)	96% (93%-99%)	66% (59%-75%)	86% (80%-92%)	46% (37%-57%)	80% (72%-88%)	<0.001
	**CNLC stage I (985, 147, NA/NA)**		93% (91%-95%)	96% (95%-98%)	83% (79%-87%)	90% (87%-93%)	74% (69%-79%)	84% (79%-88%)	0.002
	**MVI**	**Absent [639 (64.87), 60, NA/NA]**	97% (95%-99%)	96% (94%-99%)	91% (88%-95%)	93% (90%-96%)	88% (83%-92%)	86% (80%-91%)	0.841
		**Present [346 (35.13), 87, 32/NA]**	86% (81%-91%)	96% (93%-99%)	67% (59%-76%)	85% (79%-91%)	47% (38%-59%)	80% (73%-88%)	<0.001
	**CNLC stage II (71, 15, 27/NA)**		77% (62%-95%)	92% (85%-100%)	60% (43%-85%)	92% (85%-100%)	48% (30%-77%)	83% (66%-100%)	0.002
	**MVI**	**Absent[33 (46.48), 6, NA/NA]**	75% (54%-100%)	89% (75%-100%)	64% (41%-100%)	89% (75%-100%)	64% (41%-100%)	89% (75%-100%)	0.087
		**Present[38 (53.32), 9, 26/NA]**	78% (59%-100%)	96% (88%-100%)	53% (29%-98%)	96% (88%-100%)	27% (8.5%-84%)	72% (41%-100%)	0.002

**PSM**, Propensity score matching; **DFS**, Disease-free survival; **OS**, Overall survival; **MVI**, Microvascular invasion; **TACE**, Transarterial chemoembolization; **CNLC**, China liver cancer

## Abbreviations

MVI: Microvascular invasion
HCC: Hepatocellular carcinoma
TACE: Transarterial chemoembolization
PSM: Propensity Score Matching
DFS: Disease-free survival
OS: Overall survival
CNLC: Chinese liver cancer
CT: Computed tomography
MRI: Magnetic resonance imaging
SD: Standard deviation
IQR: Quartile distance
HBV: Hepatitis B virus
AFP: Alpha-fetoprotein
ALT: Alanine aminotransferase
AST: Aspartate aminotransferase
GGT: Gamma-glutamyltransferase
ALP: Alkaline phosphatase
ALB: Albumin
TB: Total bilirubin
WBC: White blood cell
CR: Creatinine
PT: Prothrombin time
NLR: Neutrophil-to-lymphocyte ratio
LMR: Lymphocyte-to-monocyte ratio
PLR: Platelet-to-lymphocyte ratio
HBV: Hepatitis B virus
