# Supplementary material for: Survival effects of postoperative adjuvant TACE in early-HCC patients with microvascular invasion: A multicenter propensity score matching
Source: J Cancer. 2024 Jan 1;15(1):68–78. doi: 10.7150/jca.87435 (PMC10751667; doi:10.7150/jca.87435)
Supplement: Supplementary file 1 — Supplementary figures and tables. [file jcav15p0068s1.pdf]

Table S1 Clinical characteristics of patients without MVI who underwent adjuvant TACE or not before PSM

Clinical characteristics		MVI absent			
		Total (n = 815)	Adjuvant TACE		P
			No (n = 431)	Yes (n = 384)	
**Age (years)**		57.00 (49.00, 65.00)	58.00 (48.00, 66.00)	56.00 (49.00, 64.00)	0.102
**AFP (ng/mL)**		17.47 (4.30, 186.25)	16.20 (4.25, 143.00)	19.19 (4.40, 264.10)	0.101
**ALT (U/L)**		30.00 (21.10, 44.67)	29.00 (20.73, 42.32)	31.39 (21.68, 47.00)	0.111
**AST (U/L)**		32.57 (25.71, 44.81)	31.22 (25.00, 44.00)	33.05 (26.00, 45.95)	0.079
**GGT (U/L)**		44.45 (27.00, 81.95)	46.00 (25.42, 81.39)	42.51 (27.36, 83.12)	0.995
**ALP (U/L)**		91.00 (72.81, 115.44)	89.00 (70.28, 112.00)	94.95 (75.00, 118.66)	0.030
**ALB (g/L)**		41.56 (38.41, 44.25)	41.20 (38.20, 44.00)	41.92 (38.70, 44.50)	0.109
**TB (mol/L)**		14.48 (10.73, 19.80)	13.60 (10.40, 19.80)	15.20 (11.10, 19.82)	0.058
**WBC (10^9/L)**		5.24 (4.16, 6.47)	5.24 (4.11, 6.48)	5.26 (4.22, 6.44)	0.628
**CR (μmol/L)**		73.00 (62.20, 83.62)	73.30 (62.92, 82.86)	72.38 (61.80, 83.84)	0.581
**PT (s)**		11.80 (11.20, 12.60)	11.80 (11.30, 12.50)	11.80 (11.20, 12.60)	0.984
**NLR**		2.10 (1.52, 3.01)	1.95 (1.43, 2.81)	2.20 (1.64, 3.30)	*<0.001*
**LMR**		3.79 (2.80, 5.10)	3.90 (2.90, 5.18)	3.62 (2.72, 5.00)	0.132
**PLR**		101.50 (78.13, 140.36)	96.69 (73.49, 136.10)	108.47 (83.88, 143.39)	*<0.001*
**Operation time (mins)**		202.00 (150.00, 260.00)	200.00 (150.00, 260.00)	210.00 (165.00, 271.25)	0.066
**Tumor diameter (mm)**		33.00 (22.00, 53.00)	33.00 (22.00, 51.00)	34.00 (22.00, 55.00)	0.197
**Gender [n(%)]**	**male**	674 (82.70)	362 (83.99)	312 (81.25)	0.347
	**female**	141 (17.30)	69 (16.01)	72 (18.75)	
**HBV [n(%)]**	**Negative**	113 (13.87)	62 (14.39)	51 (13.28)	0.724
	**Positive**	702 (86.13)	369 (85.61)	333 (86.72)	
**Child–Pugh classification [n(%)]**	**A**	782 (95.95)	414 (96.06)	368 (95.83)	1.000
	**B**	33 (4.05)	17 (3.94)	16 (4.17)	
**Liver cirrhosis [n(%)]**	**No**	219 (26.87)	119 (27.61)	100 (26.04)	0.671
	**Yes**	596 (73.13)	312 (72.39)	284 (73.96)	
**Tumor number [n(%)]**	**single**	760 (93.25)	407 (94.43)	353 (91.93)	0.200
	**multiple**	55 (6.75)	24 (5.57)	31 (8.07)	
**Tumor location [n(%)]**	**left**	249 (30.55)	137 (31.79)	112 (29.17)	0.702
	**right**	534 (65.52)	278 (64.50)	256 (66.67)	
	**double**	32 (3.93)	16 (3.71)	16 (4.17)	
**Tumor margin [n(%)]**	**Non-smooth**	128 (15.71)	68 (15.78)	60 (15.62)	1.000
	**Smooth**	687 (84.29)	363 (84.22)	324 (84.38)	
**Anatomical liver resection [n(%)]**	**No**	290 (35.58)	165 (38.28)	125 (32.55)	0.103
	**Yes**	525 (64.42)	266 (61.72)	259 (67.45)	
**Laparoscopic surgery [n(%)]**	**No**	425 (52.15)	223 (51.74)	202 (52.60)	0.860
	**Yes**	390 (47.85)	208 (48.26)	182 (47.40)	
**Satellite nodules [n(%)]**	**Negative**	759 (93.13)	406 (94.20)	353 (91.93)	0.254
	**Positive**	56 (6.87)	25 (5.80)	31 (8.07)	
**Differentiation [n(%)]**	**High-medium**	735 (90.18)	391 (90.72)	344 (89.58)	0.670
	**Low**	80 (9.82)	40 (9.28)	40 (10.42)	

PSM, Propensity score matching; **MVI**, Microvascular invasion; **TACE**, Transarterial chemoembolization; **AFP**, Alpha-fetoprotein; **ALT**, Alanine aminotransferase; **AST**, Aspartate aminotransferase; **GGT**, Gamma-glutamyltransferase; **ALP**, Alkaline phosphatase; **ALB,** Albumin; **TB**, Total bilirubin; **WBC**, White blood cell; **CR**, Creatinine; **PT**, Prothrombin time; **NLR**, Neutrophil-to-lymphocyte ratio; **LMR**, Lymphocyte-to-monocyte ratio; **PLR**, Platelet-to-lymphocyte ratio; **HBV**, Hepatitis B virus

Table S2 Clinical characteristics of MVI patients who underwent adjuvant TACE or not before PSM

Clinical characteristics		MVI present			
		Total (n = 557)	Adjuvant TACE		P
			No (n = 229)	Yes (n = 328)	
**Age (years)**		55.00 (46.00, 64.00)	55.00 (46.00, 65.00)	55.00 (46.00, 63.25)	0.919
**AFP (ng/mL)**		224.50 (16.40, 1000.00)	227.00 (18.32, 1000.00)	214.30 (16.05, 1000.00)	0.867
**ALT (U/L)**		31.00 (22.12, 47.00)	28.51 (21.00, 45.00)	33.86 (23.00, 48.79)	0.044
**AST (U/L)**		38.89 (28.07, 56.37)	35.00 (27.00, 53.62)	41.70 (29.20, 59.73)	0.010
**GGT (U/L)**		63.71 (35.23, 118.00)	61.11 (37.00, 118.24)	67.72 (35.11, 117.25)	0.946
**ALP (U/L)**		100.00 (78.02, 129.12)	99.00 (76.70, 132.50)	100.00 (80.00, 127.25)	0.777
**ALB (g/L)**		40.80 (37.80, 43.40)	39.90 (37.30, 42.86)	41.12 (38.00, 43.60)	0.006
**TB (mol/L)**		14.80 (10.94, 19.70)	15.09 (10.50, 19.95)	14.60 (11.18, 19.31)	0.933
**WBC (10^9/L)**		5.33 (4.38, 6.51)	5.53 (4.42, 6.80)	5.29 (4.33, 6.46)	0.226
**CR (μmol/L)**		73.10 (62.35, 82.23)	72.00 (61.80, 80.00)	73.89 (63.45, 83.43)	0.055
**PT (s)**		12.00 (11.40, 12.60)	12.00 (11.40, 12.60)	11.90 (11.40, 12.60)	0.699
**NLR**		2.32 (1.69, 3.360)	2.40 (1.74, 3.58)	2.27 (1.61, 3.27)	0.271
**LMR**		3.240 (2.440, 4.560)	3.24 (2.50, 4.60)	3.25 (2.43, 4.48)	0.773
**PLR**		115.79 (88.50, 165.04)	111.65 (88.10, 156.30)	118.03 (90.21, 170.09)	0.118
**Operation time (mins)**		230.00 (180.00, 288.00)	225.00 (180.00, 295.00)	235.00 (180.00, 285.00)	0.717
**Tumor diameter (mm)**		57.00 (38.00, 82.00)	50.00 (34.00, 73.00)	63.50 (41.00, 88.00)	0.001
**Gender [n(%)]**	**male**	479 (86.00)	194 (84.72)	285 (86.89)	0.546
	**female**	78 (14.00)	35 (15.28)	43 (13.11)	
**HBV [n(%)]**	**Negative**	68 (12.21)	34 (14.85)	34 (10.37)	0.145
	**Positive**	489 (87.79)	195 (85.15)	294 (89.63)	
**Child–Pugh classification [n(%)]**	**A**	529 (94.97)	211 (92.14)	318 (96.95)	0.018
	**B**	28 (5.03)	18 (7.86)	10 (3.05)	
**Liver cirrhosis [n(%)]**	**No**	129 (23.16)	50 (21.83)	79 (24.09)	0.605
	**Yes**	428 (76.84)	179 (78.17)	249 (75.91)	
**Tumor number [n(%)]**	**single**	463 (83.12)	185 (80.79)	278 (84.76)	0.264
	**multiple**	94 (16.88)	44 (19.21)	50 (15.24)	
**Tumor location [n(%)]**	**left**	178 (31.96)	73 (31.88)	105 (32.01)	0.449
	**right**	346 (62.12)	139 (60.70)	207 (63.11)	
	**double**	33 (5.92)	17 (7.42)	16 (4.88)	
**Tumor margin [n(%)]**	**Non-smooth**	201 (36.09)	88 (38.43)	113 (34.45)	0.383
	**Smooth**	356 (63.91)	141 (61.57)	215 (65.55)	
**Anatomical liver resection [n(%)]**	**No**	143 (25.67)	65 (28.38)	78 (23.78)	0.261
	**Yes**	414 (74.33)	164 (71.62)	250 (76.22)	
**Laparoscopic surgery [n(%)]**	**No**	374 (67.15)	157 (68.56)	217 (66.16)	0.616
	**Yes**	183 (32.85)	72 (31.44)	111 (33.84)	
**Satellite nodules [n(%)]**	**Negative**	439 (78.82)	165 (72.05)	274 (83.54)	0.002
	**Positive**	118 (21.18)	64 (27.95)	54 (16.46)	
**Differentiation [n(%)]**	**High-medium**	420 (75.40)	173 (75.55)	247 (75.30)	1.000
	**Low**	137 (24.60)	56 (24.45)	81 (24.70)	

PSM, Propensity score matching; **MVI**, Microvascular invasion; **TACE**, Transarterial chemoembolization; **AFP**, Alpha-fetoprotein; **ALT**, Alanine aminotransferase; **AST**, Aspartate aminotransferase; **GGT**, Gamma-glutamyltransferase; **ALP**, Alkaline phosphatase; **ALB,** Albumin; **TB**, Total bilirubin; **WBC**, White blood cell; **CR**, Creatinine; **PT**, Prothrombin time; **NLR**, Neutrophil-to-lymphocyte ratio; **LMR**, Lymphocyte-to-monocyte ratio; **PLR**, Platelet-to-lymphocyte ratio; **HBV**, Hepatitis B virus

Table S3 DFS and OS at 1, 2, and 3 years for different subgroups of population who received adjuvant TACE before PSM

Characteristics [Number (%), Event, Median time (months)]			1 year		2 year		3 year		P
			Adjuvant TACE						
			No	Yes	No	Yes	No	Yes	
DFS	All patients (1372, 462, NA/NA)		74% (70%-77%)	81% (78%-84%)	62% (58%-66%)	69% (66%-73%)	55% (51%-60%)	63% (58%-67%)	0.001
	MVI	Absent [815 (59.40), 173, NA/NA]	88% (85%-91%)	92% (89%-95%)	78% (74%-83%)	82% (78%-86%)	70% (65%-76%)	76% (71%-82%)	0.065
		Present [557 (40.60), 289, 12/28]	46% (40%-54%)	68% (63%-73%)	29% (23%-38%)	54% (48%-60%)	23% (16%-34%)	45% (38%-52%)	<0.001
	CNLC stage I (1253, 401, NA/NA)		76% (72%-79%)	82% (79%-85%)	64% (60%-69%)	71% (67%-75%)	57% (52%-62%)	64% (60%-69%)	0.002
	MVI	Absent [778 (62.09), 162, NA/NA]	88% (85%-91%)	92% (90%-95%)	78% (74%-83%)	82% (78%-87%)	70% (64%-76%)	77% (72%-83%)	0.041
		Present [475 (37.91), 239, 12/29]	48% (42%-56%)	69% (64%-75%)	31% (24%-40%)	55% (49%-62%)	25% (17%-36%)	47% (40%-56%)	<0.001
	CNLC stage II (119, 61, 12/29)		47% (34%-64%)	67% (56%-79%)	35% (23%-54%)	56% (45%-70%)	35% (23%-54%)	47% (36%-62%)	0.015
	MVI	Absent[37 (31.09), 11, NA/NA]	82% (62%-100%)	84% (70%-100%)	72% (49%-100%)	75% (60%-95%)	72% (49%-100%)	70% (54%-92%)	0.806
		Present[82 (68.91), 50, 7/22]	35% (22%-56%)	56% (43%-74%)	21% (9.5%-47%)	44% (30%-63%)	NA	31% (18%-53%)	0.006
OS	All patients (1372, 211, NA/NA)		91% (89%-93%)	97% (96%-98%)	81% (77%-84%)	89% (87%-92%)	71% (66%-76%)	84% (81%-88%)	<0.001
	MVI	Absent [815 (59.40), 73, NA/NA]	96% (94%-98%)	98% (96%-99%)	91% (87%-94%)	95% (93%-97%)	84% (80%-89%)	90% (86%-94%)	0.057
		Present [557 (40.60), 138, 30/NA]	81% (76%-86%)	96% (94%-98%)	61% (54%-69%)	82% (77%-87%)	39% (30%-51%)	77% (72%-83%)	<0.001
	CNLC stage I (n = 1253, 180, NA/NA)		92% (90%-95%)	98% (96%-99%)	83% (80%-87%)	89% (87%-92%)	73% (69%-78%)	84% (80%-88%)	<0.001
	MVI	Absent [778 (62.09), 68, NA/NA]	97% (95%-99%)	98% (96%-99%)	91% (88%-94%)	95% (92%-98%)	85% (80%-90%)	89% (85%-94%)	0.148
		Present [475 (37.91), 112, 32/NA]	83% (77%-88%)	97% (95%-99%)	65% (54%-74%)	82% (76%-87%)	42% (32%-55%)	77% (71%-84%)	<0.001
	CNLC stage II (n = 119, 31, 24/NA)		72% (60%-86%)	94% (88%-100%)	47% (33%-67%)	88% (80%-97%)	39% (25%-61%)	85% (76%-96%)	<0.001
	MVI	Absent[37 (31.09), 5, NA/NA]	74% (53%-100%)	100% (100%-100%)	63% (40%-100%)	96% (88%-100%)	63% (40%-100%)	96% (88%-100%)	0.010
		Present[82 (68.91), 26, 20/NA]	72% (58%-88%)	90% (81%-100%)	40% (24%-67%)	82% (70%-97%)	27% (12%-57%)	77% (62%-95%)	<0.001

PSM, Propensity score matching; **DFS**, Disease-free survival; **OS**, Overall survival; **MVI**, Microvascular invasion; **TACE**, Transarterial chemoembolization; **CNLC**, *China liver cancer*

Fig.S1

Schematic diagram of baseline sampling site of a liver tumor specimen (A). I, II, III, IV: Junction of carcinoma and para carcinoma tissues corresponding to clockwise 12, 3, 6 and 9; V: Tumor region; VI: Adjacent para carcinoma liver tissue region; VII: Distant para carcinoma liver tissue region. MVI (black arrow) can be seen in adjacent liver tissue adjacent to cancer [hematoxylin and eosin stain, ×100 (B), ×200 (C)].

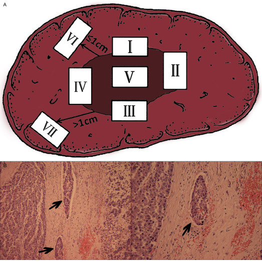

MVI, Microvascular invasion

Fig.S2 Forest plot of univariate and multivariate Cox regression analysis of DFS (A) and OS (B) after hepatectomy in HCC patients before PSM.

A

◆ Univariable ◆ Multivariable

Characteristics(Before PSM)	Univariable HR(95%CI)	Univariable P Value	Multivariable HR(95%CI)	Multivariable P Value
Age	0.996(0.988-1.003)	0.269		
AFP	1.000(1.000-1.000)	<0.001	1.000(1.000-1.000)	<0.001
ALT	1.002(1.001-1.004)	0.001	1.002(0.999-1.005)	0.244
AST	1.003(1.002-1.004)	<0.001	0.999(0.996-1.002)	0.512
GGT	1.001(1.000-1.001)	<0.001	1.000(1.000-1.001)	0.338
ALP	1.000(1.000-1.001)	0.251		
ALB	0.966(0.948-0.985)	0.001	0.982(0.962-1.003)	0.094
TB	1.004(0.997-1.010)	0.265		
WBC	1.004(0.985-1.024)	0.679		
CR	0.997(0.992-1.002)	0.221		
PT	1.030(0.961-1.104)	0.405		
NLR	1.012(0.987-1.036)	0.353		
LMR	1.010(0.999-1.022)	0.079		
PLR	1.001(1.000-1.002)	0.116		
Operation time	1.001(1.000-1.002)	0.069		
Tumor diameter	1.011(1.009-1.014)	<0.001	1.006(1.003-1.009)	<0.001
**Gender**				
Male				
Female	0.886(0.685-1.147)	0.358		
**HBV**				
Negative				
Positive	1.139(0.857-1.513)	0.370		
**Child-Pugh classification**				
A				
B	1.176(0.773-1.788)	0.450		
**Liver cirrhosis**				
No				
Yes	1.141(0.923-1.411)	0.222		
**Tumor number**				
Single				
Multiple	1.766(1.371-2.274)	<0.001	1.316(1.011-1.711)	0.041
**Tumor location**				
Left				
Right	0.967(0.793-1.180)	0.744		
Double	1.091(0.703-1.694)	0.697		
**Tumor margin**				
Non-smooth				
Smooth	0.709(0.578-0.870)	0.001	1.063(0.855-1.321)	0.583
**Anatomical liver resection**				
No				
Yes	1.382(1.124-1.701)	0.002	1.264(1.024-1.561)	0.029
**Laparoscopic surgery**				
No				
Yes	0.737(0.608-0.893)	0.002	0.911(0.746-1.113)	0.361
**Satellite nodules**				
Negative				
Positive	2.762(2.207-3.456)	<0.001	1.803(1.429-2.277)	<0.001
**Differentiation**				
High-medium				
Low	2.860(2.322-3.523)	<0.001	2.133(1.718-2.647)	<0.001
**MVI**				
Absent				
Present	3.616(2.989-4.376)	<0.001	2.708(2.177-3.371)	<0.001
**Adjuvant TACE**				
No				
Yes	0.738(0.614-0.855)	0.001	0.513(0.424-0.621)	<0.001

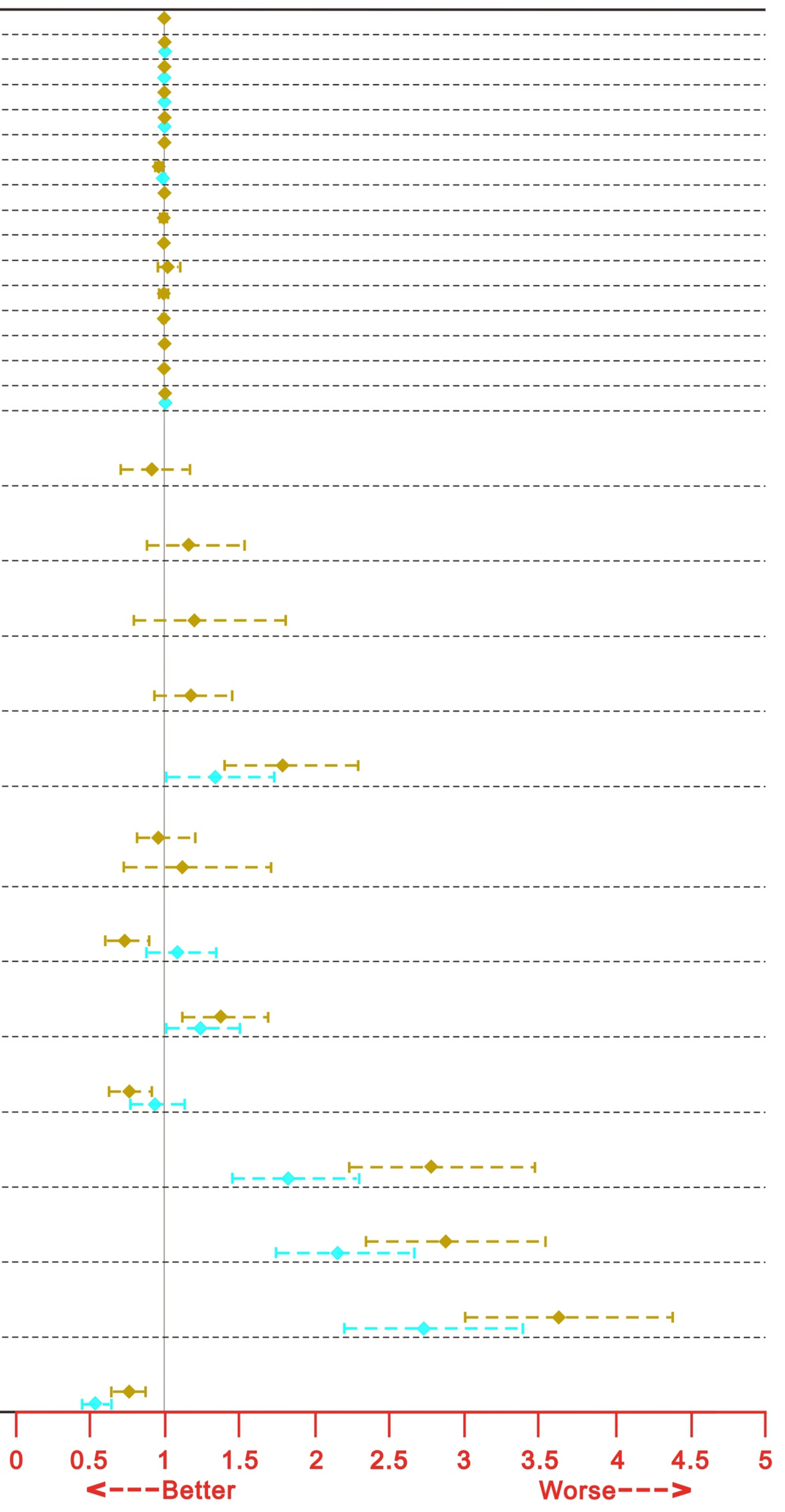

B

◆ Univariable ◆ Multivariable

Characteristics(Before PSM)	Univariable HR(95%CI)	Univariable P Value	Multivariable HR(95%CI)	Multivariable P Value
Age	1.006(0.994-1.017)	0.342		
AFP	1.000(1.000-1.000)	<0.001	1.000(1.000-1.000)	0.288
ALT	1.003(1.001-1.005)	0.007	1.001(0.996-1.006)	0.718
AST	1.003(1.002-1.005)	<0.001	1.000(0.995-1.004)	0.934
GGT	1.001(1.000-1.001)	0.007	1.000(0.999-1.000)	0.289
ALP	1.001(1.000-1.002)	<0.001	1.001(1.000-1.002)	0.169
ALB	0.945(0.919-0.971)	<0.001	0.972(0.941-1.003)	0.076
TB	1.010(1.004-1.017)	0.001	1.007(0.998-1.015)	0.129
WBC	0.998(0.956-1.042)	0.936		
CR	0.994(0.986-1.002)	0.149		
PT	1.071(0.975-1.176)	0.152		
NLR	1.023(0.990-1.057)	0.175		
LMR	1.018(1.007-1.028)	0.001	1.007(0.998-1.016)	0.129
PLR	1.001(0.999-1.003)	0.229		
Operation time	1.002(1.000-1.003)	0.016	1.001(0.999-1.002)	0.426
Tumor diameter	1.012(1.008-1.016)	<0.001	1.009(1.005-1.014)	<0.001
**Gender**				
Male				
Female	0.979(0.679-1.414)	0.912		
**HBV**				
Negative				
Positive	0.916(0.616-1.364)	0.667		
**Child-Pugh classification**				
A				
B	2.302(1.452-3.650)	<0.001	1.405(0.787-2.507)	0.250
**Liver cirrhosis**				
No				
Yes	2.088(1.446-3.015)	<0.001	2.078(1.417-3.047)	<0.001
**Tumor number**				
Single				
Multiple	1.850(1.293-2.649)	0.001	1.214(0.828-1.781)	0.321
**Tumor location**				
Left				
Right	0.896(0.670-1.200)	0.463		
Double	1.053(0.557-1.990)	0.874		
**Tumor margin**				
Non-smooth				
Smooth	0.632(0.471-0.848)	0.002	0.870(0.634-1.196)	0.392
**Anatomical liver resection**				
No				
Yes	1.299(0.961-1.755)	0.088		
**Laparoscopic surgery**				
No				
Yes	0.596(0.441-0.805)	0.001	0.839(0.611-1.150)	0.275
**Satellite nodules**				
Negative				
Positive	3.117(2.284-4.252)	<0.001	2.131(1.527-2.973)	<0.001
**Differentiation**				
High-medium				
Low	2.745(2.031-3.712)	<0.001	1.926(1.403-2.644)	<0.001
**MVI**				
Absent				
Present	3.590(2.699-4.776)	<0.001	2.373(1.704-3.306)	<0.001
**Adjuvant TACE**				
No				
Yes	0.510(0.386-0.674)	<0.001	0.369(0.275-0.494)	<0.001

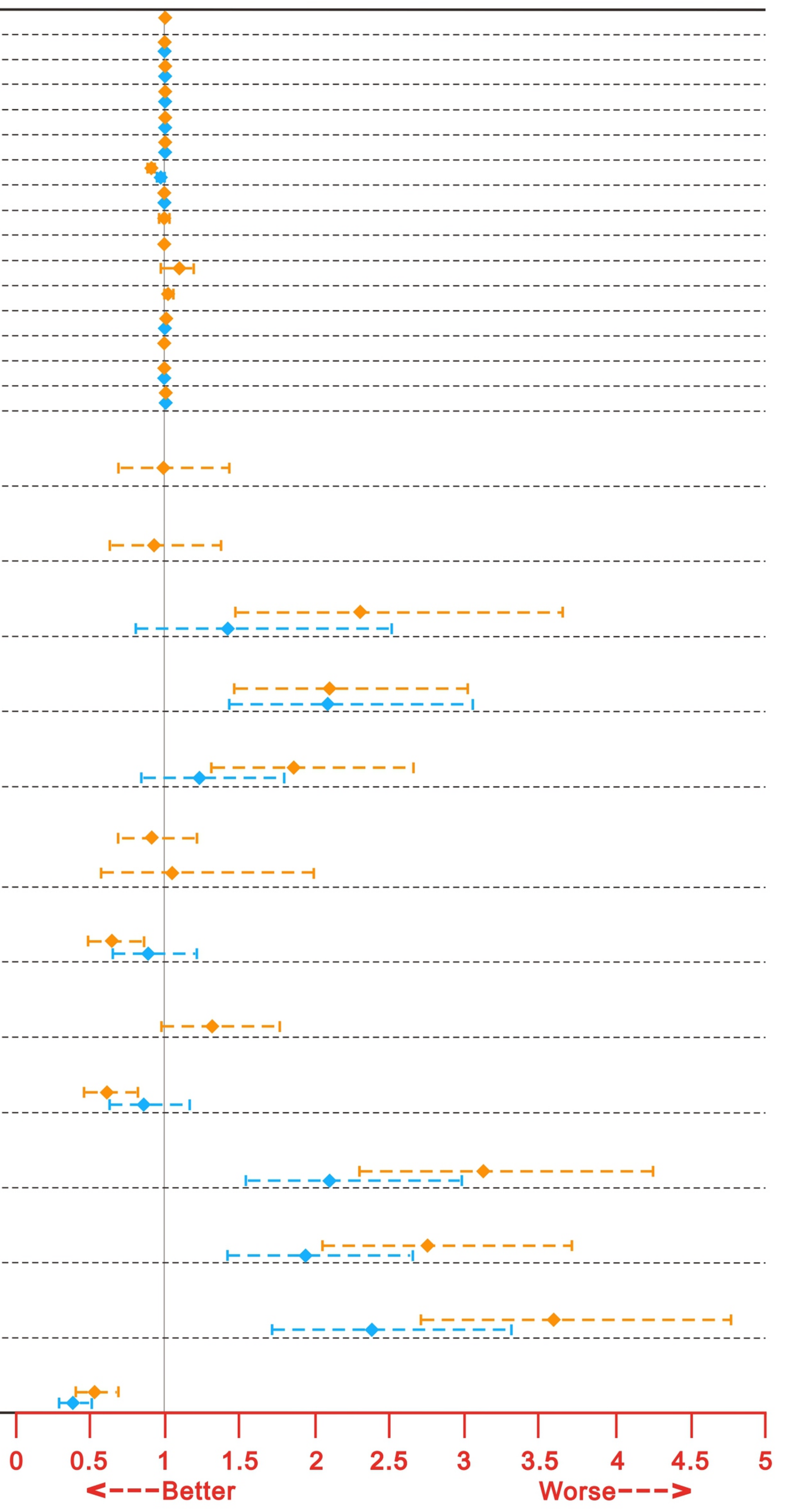

HCC, Hepatocellular carcinoma; PSM, Propensity score matching; DFS, Disease-free survival; OS, Overall survival; MVI, Microvascular invasion; TACE, Transarterial chemoembolization; HR, Hazard?ratio; CI, Confidence interval; AFP, Alpha-fetoprotein; ALT, Alanine aminotransferase; AST, Aspartate aminotransferase; GGT, Gamma-glutamyltransferase; ALP, Alkaline?phosphatase; Alb, Albumin; TB, Total bilirubin; WBC, White blood cell; CR, Creatinine; PT, Prothrombin time; NLR, Neutrophil-to-lymphocyte ratio; LMR, Lymphocyte-to-monocyte ratio; PLR, Platelet-to-lymphocyte ratio; HBV, Hepatitis B virus

Fig.S3 Kaplan-meier analysis of DFS (A) and OS (B) in HCC patients with or without MVI before PSM; Kaplan-meier analysis of DFS (C) and OS (D) in HCC patients receiving adjuvant TACE or not before PSM; Subgroup Kaplan-meier analysis of DFS (E) and OS (F) in patients with and without MVI receiving adjuvant TACE before PSM.

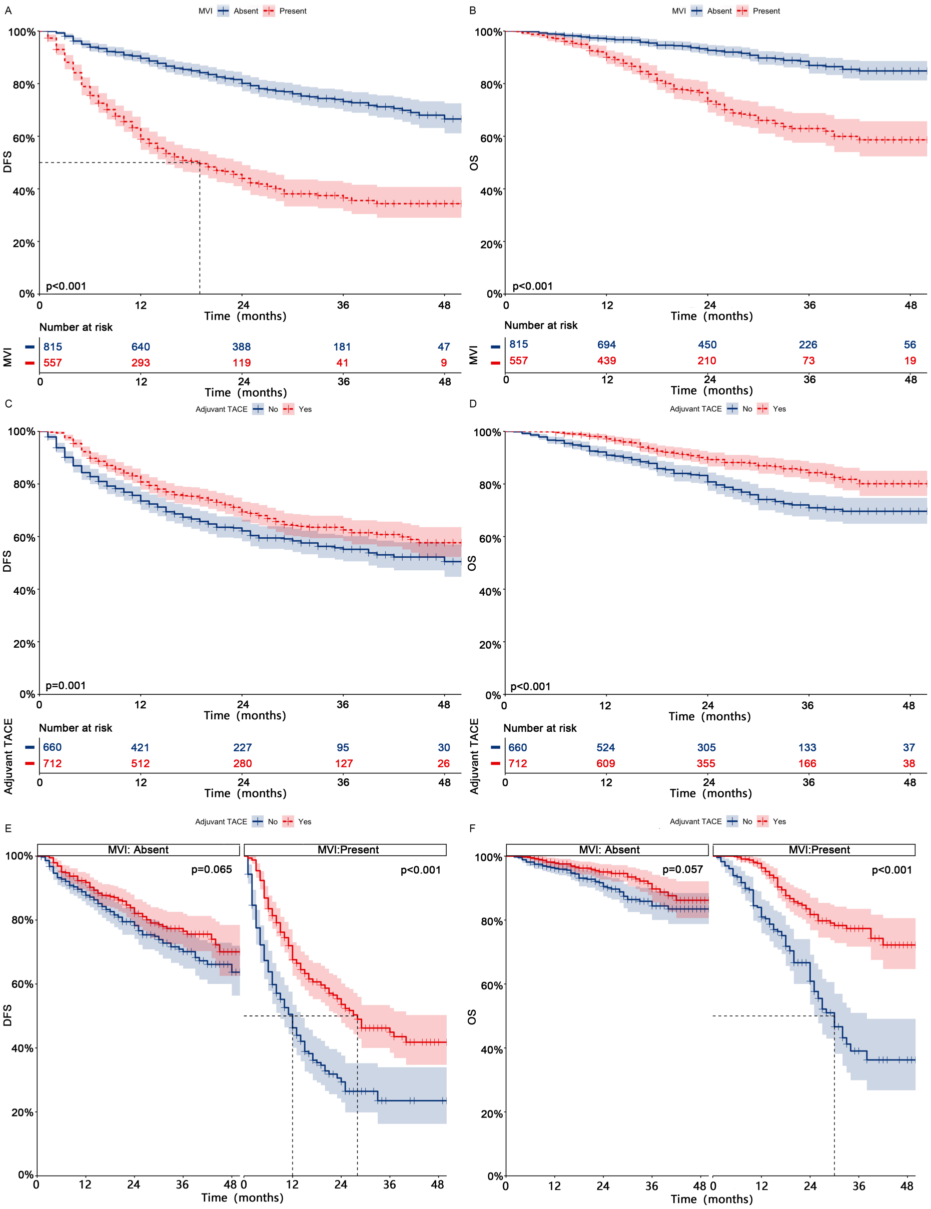

HCC, Hepatocellular carcinoma; PSM, Propensity score matching; DFS, Disease-free survival; OS, Overall survival; MVI, Micro-vascular invasion; TACE, Transarterial chemoembolization

Fig.S4 Kaplan-meier analysis of DFS (A) and OS (B) for patients with different CNLC stages before PSM; Subgroup Kaplan-meier analysis of DFS (C) and OS (D) for patients with different CNLC stages receiving adjuvant TACE before PSM.

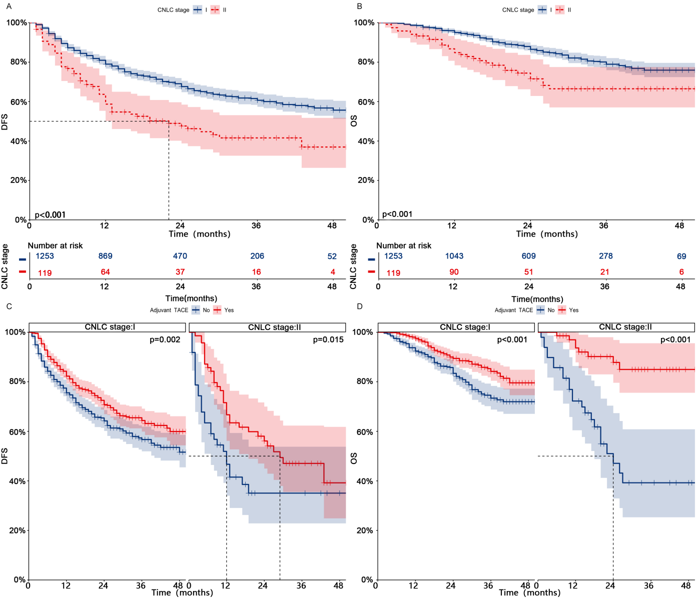

PSM, Propensity score matching; DFS, Disease-free survival; OS, Overall survival; TACE, Transarterial chemoembolization; CNLC, China liver cancer

Fig.S5 Subgroup Kaplan-meier analysis of DFS (AC) and OS (BD) for MVI patients in different CNLC stages who received adjuvant TACE before PSM.

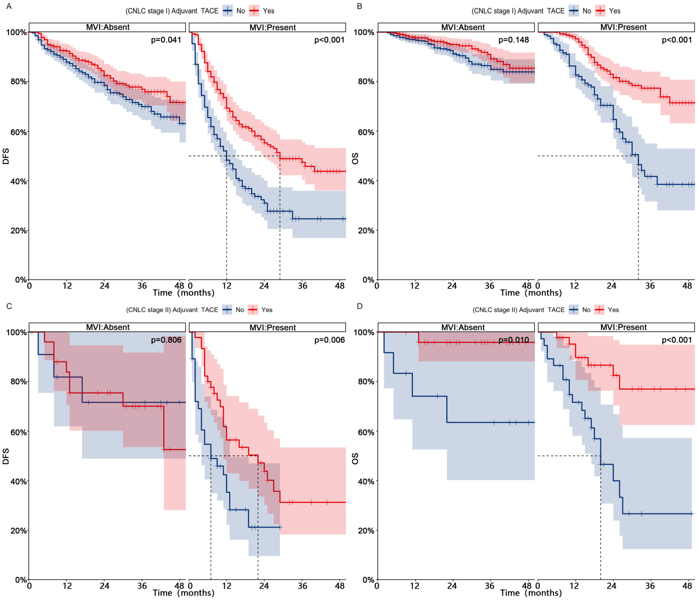

PSM, Propensity score matching; DFS, Disease-free survival; OS, Overall survival; MVI, Micro-vascular invasion; TACE, Transarterial chemoembolization; CNLC, China liver cancer
